# Development and preliminary evaluation of a German questionnaire assessing attitudes toward hearing aids and behavioral intention

**DOI:** 10.1186/s12913-026-15549-0

**Published:** 2026-09-13

**Authors:** Corinna Mathern, Katrin Reimann, Rainer M. Weiß, Johanna R. Rusche, Boris A. Stuck, Kruthika Thangavelu

**Affiliations:** Department of Otorhinolaryngology, Head and Neck Surgery, Faculty of Medicine, Philipps-Universität Marburg, Baldingerstrasse, Marburg, 35047 Germany

**Keywords:** Hearing aids, Attitudes, Behavioral intention, Psychometrics, Questionnaire development, Stigma

## Abstract

**Background:**

Hearing aid uptake remains suboptimal despite well-established clinical benefits, suggesting that psychosocial factors influence hearing rehabilitation decisions. This study aimed to develop and perform a preliminary psychometric evaluation of a German-language questionnaire assessing attitudes toward hearing aids and behavioral intention toward hearing aid use.

**Methods:**

A cross-sectional survey was conducted among German-speaking adults with and without self-reported hearing loss. An existing English-language questionnaire was substantially adapted for the German healthcare system through expert review, psychometric evaluation, and pilot revision before final administration. Preliminary psychometric evaluation included assessment of internal consistency and exploratory factor analysis. Associations between attitudes, behavioral intention, and hearing aid use were examined using correlation and regression analyses.

**Results:**

The overall attitude scale demonstrated acceptable internal consistency (Cronbach’s α = 0.77), whereas the behavioral intention scale demonstrated good internal consistency (α = 0.86). Exploratory factor analysis supported a four-factor structure, although several items demonstrated cross-loadings, suggesting that further refinement of the questionnaire is warranted. The study included 159 adults (18–96 years), of whom 48.4% reported hearing loss. General attitude was strongly associated with behavioral intention (*r* = 0.63, *p* < 0.001) and explained approximately 40% of its variance (R² = 0.40). Among attitudinal domains, expected benefit and effort (*r* = 0.66) and stigma (*r* = 0.65) showed the strongest associations with behavioral intention. Participants with hearing loss who used hearing aids demonstrated significantly more favorable attitudes and stronger behavioral intention than non-users (both *p* < 0.001).

**Conclusions:**

This study describes the development and preliminary psychometric evaluation of a German-language questionnaire assessing attitudes toward hearing aids and behavioral intention. The findings provide preliminary evidence supporting the questionnaire’s reliability and structural validity while highlighting the need for further psychometric validation in independent populations. Stigma-related beliefs and expectations regarding hearing aid benefit emerged as the attitudinal domains most strongly associated with behavioral intention and may represent important targets for interventions to improve hearing aid uptake.

**Supplementary Information:**

The online version contains supplementary material available at https://doi.org/10.1186/s12913-026-15549-0.

## Background

Hearing loss is highly prevalent worldwide and is associated with reduced quality of life, social isolation, and cognitive decline [41]. Despite well-established benefits of hearing aid use, uptake remains consistently low across countries [6]. Hearing aid use among adults aged over 50 years with mild to moderate hearing loss in the United States range from 4.3% to 22.1% [8]. Similarly, European data indicate adoption rates between 14.7% and 27.1% among adults with mild hearing loss in Germany, France, and the United Kingdom [5]. Despite this heterogeneity, the overall picture clearly indicates a substantial and persistent underutilization of hearing aids.

In Germany, population-based data on hearing aid uptake remain limited but suggest improving adoption rates over recent years. According to the EuroTrak Hearing Study 2025, 47% of adults with self-reported hearing loss reported using hearing aids, compared with 41.1% in 2022 and 36.9% in 2018 [4]. Although these figures compare favorably with those reported in several international studies, more than half of individuals with self-reported hearing loss still do not use hearing aids. Understanding the factors that influence hearing aid uptake therefore remains an important public health objective.

Multiple factors have been associated with hearing aid uptake, including perceived benefit, stigma, cost, aesthetics, social influences, health literacy, and interactions with healthcare professionals. Although demographic and clinical characteristics have been investigated extensively, psychosocial determinants appear to play an important role in the decision to adopt hearing aids. For example, Tahden et al. [38] reported associations between hearing aid use and personality characteristics, whereas Knudsen et al. [22] found no consistent associations with age or sex.

Existing research has predominantly focused on hearing aid discontinuation after fitting [15, 26, 27]. Comparatively fewer studies have examined the earlier stages of help-seeking and decision-making, including why individuals delay hearing assessment, decline hearing aid fitting, or choose not to adopt hearing aids despite recognizing hearing difficulties [16, 18, 34]. Several studies have investigated attitudes toward hearing aids—including stigma, perceived benefit, aesthetic concerns, trust in healthcare providers, and financial barriers—but these constructs have generally been assessed separately or using study-specific measures, making comparisons across studies difficult [2, 10, 11, 19, 21, 24, 31, 32, 35, 36]. To our knowledge, no widely adopted German-language instrument comprehensively assesses multiple attitudinal domains toward hearing aids together with behavioral intention within a single questionnaire.

Behavioral intention is regarded as one of the strongest predictors of future health-related behavior according to the Theory of Reasoned Action, which proposes that behavior is primarily determined by an individual’s attitudes and perceived social influences. This framework has been widely applied in health behavior research and provides a useful theoretical basis for understanding hearing aid adoption. Consequently, the present questionnaire was designed to assess multiple attitudinal domains alongside behavioral intention toward hearing aid use.

The present study aimed to develop and perform a preliminary psychometric evaluation of a multidimensional German-language questionnaire assessing attitudes toward hearing aids and behavioral intention toward hearing aid use. Specifically, the study aimed to examine the associations between attitudinal domains, behavioral intention and hearing aid usage in an adult German population. By providing a standardized and theory-informed instrument, this study aims to support future research on hearing aid uptake in Germany and to facilitate cross-national comparisons in international contexts.

## Materials and methods

### Study design

An existing English-language questionnaire was substantially adapted for the German healthcare context through expert review and pilot testing. A cross-sectional survey was subsequently conducted among German-speaking adults with and without self-reported hearing loss. Preliminary psychometric evaluation included assessment of internal consistency and exploratory factor analysis. Construct validity was explored through associations between attitudes and behavioral intention and comparisons according to hearing aid use.

### Questionnaire development and initial adaptation

The initial version of the questionnaire was adapted from a doctoral dissertation by O’Shaughnessy, which described the development of an English-language questionnaire assessing attitudes toward hearing aids based on the ABC model of attitudes (affective, behavioral, and cognitive components) [33]. The author of the original dissertation was contacted to request permission for use and adaptation of the questionnaire; however, no response was received. The original instrument comprised 18 attitude statements, each paired with a corresponding behavioral intention item, and was developed using concepts derived from the Attitudes toward Loss of Hearing Questionnaire (ALHQ) and the Hearing Attitudes in Rehabilitation Questionnaire (HARQ). Thus, the initial item selection was informed by an established theoretical framework and existing hearing-related attitude measures.

To improve applicability within the German healthcare system and contemporary hearing rehabilitation practice, the questionnaire underwent substantial adaptation rather than direct translation. Original items were adapted where necessary, and additional items were developed to address topics considered relevant in the German context, including reimbursement by statutory health insurance, physician counseling, hearing aid aesthetics, and expectations regarding hearing aid benefit. This process resulted in an initial German version comprising 26 attitude items, each paired with a corresponding behavioral intention item.

Questionnaire development was primarily guided by the ABC model of attitudes, which conceptualizes attitudes as comprising affective, behavioral, and cognitive components. The Theory of Reasoned Action informed the interpretation of associations between attitudes and behavioral intention but was not used directly for item generation.

### Expert and psychometric review

The initial German questionnaire underwent expert review by two senior otologists and the head of audiology at the study institution, who were selected based on their clinical expertise in hearing rehabilitation. The questionnaire was reviewed during two discussion meetings. Feedback focused primarily on item wording, clarity, comprehensibility, and clinical applicability. Modifications were made to improve the wording and remove ambiguities.

In parallel, an experienced psychometrician independently reviewed the questionnaire and provided written feedback by email, with particular attention to the translation, questionnaire structure, item wording, and response format. This review also identified potentially confusing formulations, including double-negative wording. The psychometrician’s recommendations were subsequently discussed with the clinical experts during the review meetings and incorporated into the revised questionnaire.

### Pilot testing and final questionnaire

The revised questionnaire was subsequently pilot tested in 25 adults randomly recruited from patients and accompanying persons attending the outpatient clinic. Participants completed the questionnaire and were invited to provide additional comments through an open-ended feedback question at the end of the survey. Feedback regarding clarity, comprehensibility, and ease of completion was reviewed by the study team.

Based on the pilot feedback, the questionnaire was further streamlined. The number of paired attitudes–behavioral intention items was reduced from 26 to 21, and the original 10-point response format was replaced by a five-point response scale to improve clarity and ease of completion. The resulting final questionnaire therefore comprised 21 attitude items and 21 corresponding behavioral intention items (42 items arranged as 21 pairs). The final German questionnaire and its English-language version are provided in the Supplementary Material.

### Participants and data collection

The final questionnaire was administered as a paper-based survey at the outpatient Department of Otorhinolaryngology, Head and Neck Surgery, Marburg University Hospital. Consecutive adult patients attending the outpatient clinic, as well as accompanying adult persons present during the clinic visit, were invited to participate voluntarily. Recruitment was conducted over a three-month period.

Adults aged 18 years or older were eligible to participate regardless of hearing status or previous hearing aid experience. This heterogeneous sampling strategy was chosen to capture a broad range of attitudes toward hearing aids during the preliminary psychometric evaluation of the instrument. The questionnaire was completed anonymously. Incomplete questionnaires were excluded from the final analyses.

### Questionnaire composition and scoring

The final questionnaire developed through the stages described above consisted of two sections. The first section collected demographic and hearing-related information, including age, sex, education, self-reported hearing status, hearing aid ownership and use, and previous hearing aid experience. The second section contained the 21 attitude–behavioral intention pairs. For each attitude statement (Part A), participants indicated their level of agreement using a five-point response scale. Each attitude item was followed by a corresponding behavioral intention item (Part B), assessing the participant’s intended behavior in relation to the preceding belief statement.

Attitude items were coded so that higher scores consistently represented less favorable attitudes toward hearing aids, with reverse coding applied where necessary. Behavioral intention items were coded such that higher scores indicated lower intention to seek or use hearing aids.

Prior to psychometric evaluation, the authors provisionally assigned the attitude items to six conceptual domains based on item content, the underlying theoretical framework, and previous literature on factors influencing hearing aid uptake. These domains were developed for the present study to guide questionnaire development and exploratory analyses and did not represent an established factor structure of the original English-language questionnaire. These domains were intended to guide questionnaire development and exploratory analyses rather than to represent an established factor structure. Their empirical structure was subsequently examined using exploratory factor analysis.

### Statistical analysis

Statistical analyses were performed using Stata version 14.0 (StataCorp, College Station, TX, USA). Descriptive statistics were used to summarize participant characteristics and questionnaire responses. Continuous variables are presented as means and standard deviations (SD), and categorical variables as frequencies and percentages.

Internal consistency was evaluated using Cronbach’s alpha for the overall attitude scale, behavioral intention scale, and the six provisionally defined conceptual domains. Item-total statistics and the effect of individual item deletion on Cronbach’s alpha were examined to assess the contribution of individual items to scale reliability.

Structural validity was explored using exploratory factor analysis. Bartlett’s test of sphericity was performed to assess the suitability of the correlation matrix for factor analysis. Factors were extracted using principal axis factoring with Promax oblique rotation, as correlations between latent constructs were expected. The number of factors retained was guided by eigenvalues greater than 1, inspection of the scree plot, and interpretability of the resulting factor solution. Factor loadings ≥ 0.30 were considered meaningful. One attitude item (“I believe I can cope with my hearing loss and do not need a hearing aid”) was excluded from the primary exploratory factor analysis because it generated structurally missing responses among participants without self-reported hearing loss.

Preliminary construct validity was assessed by comparing attitude domain scores and behavioral intention scores between hearing aid users and non-users among participants with self-reported hearing loss using independent-samples *t*-tests. Associations between attitude domains and behavioral intention were examined using Pearson correlation coefficients. Effect sizes for group comparisons were calculated using Cohen’s *d*. Linear regression analyses were performed to investigate the relationship between attitude domain scores and behavioral intention while adjusting for age and hearing aid use status where appropriate. Missing data were handled according to the requirements of each statistical analysis. All statistical tests were two-sided, and a p-value < 0.05 was considered statistically significant.

## Results

### Sample characteristics

The final sample comprised 159 participants. Age ranged from 18 to 96 years (mean 46.6 ± 16.4 years). Overall, 91 participants (57.2%) identified as female, 67 (42.1%) as male, and one (0.6%) as diverse. Self-reported hearing loss was reported by 77 participants (48.4%), whereas 82 (51.6%) reported no hearing loss.

### Preliminary psychometric evaluation

#### Internal consistency

The attitude scale demonstrated acceptable internal consistency (Cronbach’s α = 0.77), whereas the behavioral intention scale demonstrated good internal consistency (Cronbach’s α = 0.86). Internal consistency estimates for the individual six provisionally defined attitudinal domains are presented in Table 1 and item-level internal consistency statistics for the 20-item attitude questionnaire is presented in Supplementary Table S2.

**Table 1:** Internal consistency of the overall questionnaire scales and provisionally defined attitudinal domains

Scale/domain	Items included in analysis, *n*	Cronbach’s α
**Overall scales**		
Overall attitude	20*	0.77
Behavioral intention	20*	0.86
**Provisional attitudinal domains†**		
Cost concerns	4	0.64
Stigma / embarrassment	4	0.76
Aesthetics & visibility	3	0.39
Social influence	3	0.42
Expected benefit & effort	5*	0.55
Trust in physicians / health system	3	0.55

The final questionnaire comprised 21 attitude items and 21 corresponding behavioral intention items (42 items in total)

* Item 3 was not applicable to participants without self-reported hearing loss and was therefore excluded from the internal consistency analyses. Accordingly, the overall attitude and behavioral intention scales comprised 20 items each, and the Expected benefit & effort domain comprised 5 items for this analysis

† The six provisional attitudinal domains comprise subsets of the attitude items and are not mutually exclusive. Items 4a and 10a were assigned to more than one domain because of their conceptual relevance to multiple constructs. Behavioral intention items were analyzed separately and were not assigned to these domains

#### Exploratory factor analysis

Bartlett’s test of sphericity was significant (χ²(190) = 727.57, *p* < 0.001), indicating that the correlation matrix was suitable for factor analysis. Exploratory factor analysis using principal axis factoring with Promax rotation was performed on the 20 attitude items after excluding one item that was not applicable to respondents without hearing loss. Based on eigenvalues greater than 1, inspection of the scree plot (Supplementary Fig. 1), and interpretability of the rotated solution, a four-factor solution was retained. The six provisional attitudinal domains had been defined by the authors of the present study based on item content, the underlying theoretical framework, and previous literature on hearing aid uptake; they were not derived from an established factor structure of the original English-language questionnaire. The exploratory factor analysis of the adapted questionnaire instead suggested a partially overlapping four-factor structure. Several items demonstrated cross-loadings and relatively high uniqueness, indicating that further refinement of the questionnaire may improve its structural validity. Rotated factor loadings are presented in Supplementary Table S1. Several items demonstrated cross-loadings and relatively high uniqueness, indicating that further refinement of the questionnaire may improve its structural validity. Rotated factor loadings are presented in Supplementary Table S1.

### Hearing aid use among participants with self-reported hearing loss

Among the 77 participants reporting hearing loss, 22 (28.6%) currently used hearing aids, seven (9.1%) owned hearing aids but did not use them, and 47 (61.0%) did not own hearing aids. One participant did not provide a response. Overall, 54 participants (70.1%) with hearing loss either did not use their hearing aids or did not own hearing aids (Table 2).

**Table 2:** Hearing aid ownership and use among participants with self-reported hearing loss

Hearing aid status	*n*	%
Uses hearing aids	22	28.6
Owns but does not use	7	9.1
Does not own hearing aids	47	61.0
Missing	1	1.3
Total	77	100

### Attitude and behavioral intention towards hearing aids

The overall attitude score was 2.65 ± 0.47, indicating moderately favorable attitudes toward hearing aids. The mean behavioral intention score was 2.28 ± 0.53, indicating a generally positive intention toward hearing-aid uptake.

Participants with and without self-reported hearing loss did not differ significantly in overall attitudes toward hearing aids (t(157) = − 0.98, *p* = 0.33) or behavioral intention (t(157) = 0.02, *p* = 0.99). Among participants with hearing loss, hearing-aid users demonstrated significantly more favorable attitudes than non-users (2.34 ± 0.40 vs. 2.73 ± 0.45; *p* < 0.001) and significantly stronger behavioral intention (1.93 vs. 2.43; *p* < 0.001). Both differences were associated with large effect sizes (attitude: Cohen’s *d* = − 0.87; intention: *d* = − 0.91).

General attitude toward hearing aids was strongly associated with behavioral intention (*r* = 0.63, *p* < 0.001). Linear regression demonstrated that attitude significantly predicted behavioral intention (B = 0.73, SE = 0.07, *p* < 0.001), explaining approximately 40% of the variance (R²=0.40).

In multivariable regression, age and hearing loss status explained 6.1% of the variance in behavioral intention (Model 1; R²=0.06). Older age was independently associated with stronger behavioral intention, whereas hearing loss status was not. After adding general attitude (Model 2), explained variance increased to 40% (ΔR²=0.34, *p* < 0.001). General attitude remained the only significant predictor, whereas age and hearing loss status were no longer independently associated with behavioral intention.

Item-level analyses (Supplementary Table S3) showed that cost concerns represented the most strongly endorsed barrier, whereas explicit stigma-related beliefs were generally weakly endorsed. Participants generally perceived hearing aids as helpful and discreet. Considerable variability was observed for items relating to social influence and physician communication.

**Fig. 1:**
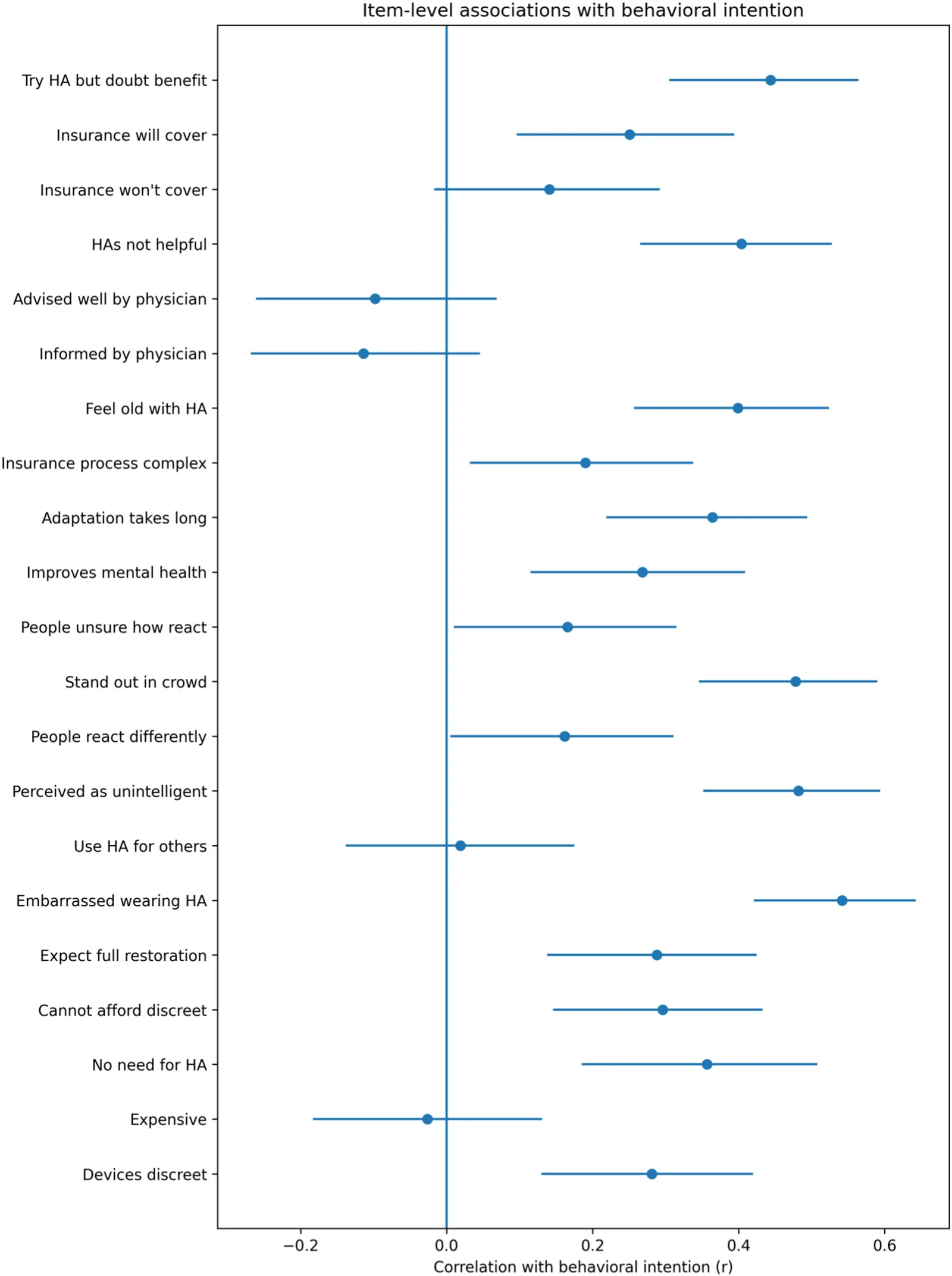
Item-level correlations between attitude items and behavioral intention toward hearing aid (HA) use. Pearson correlation coefficients (r) are shown for the association between each attitude item (Part A) and the global behavioral intention score (Part B). Error bars represent 95% confidence intervals. Positive correlation coefficients indicate that more negative attitudes toward hearing aids are associated with lower behavioral intention to seek or use hearing aids. Items are ordered according to the questionnaire sequence. Questionnaire item numbers corresponding to each statement are provided in Supplementary Table S2. Abbreviations: HA, Hearing aid; r, Pearson correlation coefficient

Item-level correlations with behavioral intention varied substantially (Fig. 1). The strongest associations were observed for stigma-related beliefs, including embarrassment, appearing unintelligent, and standing out in social situations (*r* ≈ 0.48–0.54). Beliefs regarding expected benefit also demonstrated moderate-to-strong associations (*r* ≈ 0.40–0.44), whereas physician communication and social influence showed weak or negligible associations.

### Domain based results

**Fig. 2:**
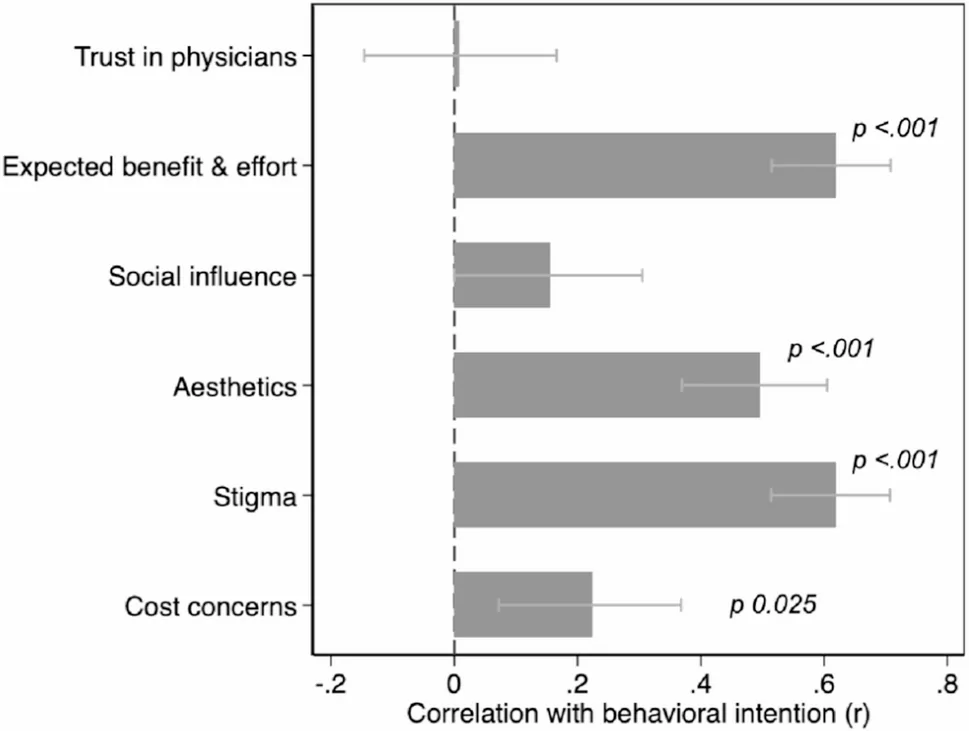
Attitudinal domain-level association with behavioral intention

Cost concerns showed the highest mean score (3.25 ± 0.83), followed by trust in physicians and the healthcare system (2.94 ± 0.98). Lower scores were observed for aesthetics and visibility (2.40 ± 0.77) and stigma/embarrassment (2.13 ± 0.92). Expected benefit and effort (*r* = 0.66) and stigma (*r* = 0.65) demonstrated the strongest associations with behavioral intention, whereas aesthetics showed a moderate association (*r* = 0.42). Cost concerns were only weakly associated with intention (*r* = 0.18), while social influence and trust in physicians were not significantly associated (Fig. 2).

Among participants with self-reported hearing loss, hearing-aid users demonstrated significantly more favorable domain scores for stigma, expected benefit and effort, and trust in physicians than non-users, with moderate-to-large effect sizes (Table 3). No significant differences were observed for cost concerns, aesthetics, or social influence.

**Table 3:** Attitudinal domain scores by hearing aid use status (with effect sizes)

Domain	Users M (SD)	Non-Users M (SD)	T test	*p*	Cohen’s d	95% CI for d
Cost concerns	3.13 (0.98)	3.37 (0.82)	−1.00	0.325	−0.27	[− 0.77, 0.22]
Stigma / embarrassment	1.60 (0.61)	2.27 (0.93)	−3.70	**< 0.001**	**−0.78**	[− 1.29, − 0.27]
Aesthetics & visibility	2.33 (0.93)	2.49 (0.74)	−0.71	0.483	−0.20	[− 0.69, 0.30]
Social influence	2.65 (0.80)	2.58 (0.95)	0.32	0.751	0.08	[− 0.43, 0.58]
Expected benefit & effort	2.29 (0.55)	2.77 (0.67)	−3.23	**0.002**	**−0.75**	[− 1.26, − 0.24]
Trust in physicians / health system	2.02 (0.93)	2.88 (1.01)	−3.51	**0.001**	**−0.87**	[− 1.39, − 0.35]

## Discussion

The present study examined attitudes toward hearing aids and their association with behavioral intention and hearing aid use in a heterogeneous adult sample. First, although overall attitudes were moderately favorable, hearing aid uptake among participants with self-reported hearing loss was low, with only 28.6% reporting current use. Second, general attitude strongly predicted behavioral intention, explaining 40% of the variance beyond age and hearing loss status. Third, domain-level analyses revealed that stigma-related beliefs and expectations regarding benefit and effort were most strongly associated with intention. Finally, among participants with hearing loss, hearing aid users demonstrated substantially more favorable attitudes than non-users, particularly in the domains of stigma, expected benefit, and trust in physicians. Importantly, these results must be interpreted within the broader context of structured hearing healthcare provision in Germany, where statutory insurance coverage, standardized fitting procedures, and quality assurance mechanisms are well established. Despite these structural supports, uptake remains limited, underscoring the decisive role of attitudinal factors.

### Preliminary psychometric evaluation

The present study included a preliminary psychometric evaluation of the newly developed German questionnaire. The overall attitude and behavioral intention scales demonstrated acceptable and good internal consistency, respectively, supporting their use for exploratory analyses. Exploratory factor analysis identified a four-factor structure, whereas the questionnaire had originally been organized into six conceptually derived domains based on theoretical considerations and previous literature.

### Questionnaire structure and internal consistency

Internal consistency varied across the theoretically derived domains. While the overall attitude and behavioral intention scales demonstrated acceptable and good reliability, respectively, several provisional domains showed lower internal consistency. This finding is consistent with the exploratory factor analysis, which suggested a partially overlapping four-factor structure rather than six clearly distinct constructs. Together, these findings indicate that some domains may require further refinement and that the latent structure of the questionnaire should be confirmed in an independent sample using confirmatory factor analysis.

### Persistent gap between hearing loss and uptake

The finding that only 28.6% of individuals with self-reported hearing loss were current hearing aid users aligns closely with international epidemiological data indicating uptake rates between 10 and 25% [22] and 20–25% [25]. Similarly, large-scale German population data from the Gutenberg Health Study indicate substantial prevalence of hearing impairment alongside incomplete hearing aid provision, even within a universal insurance framework [12]. These findings underscore the substantial gap between perceived hearing impairment and hearing-aid uptake, indicating persistent underutilization of hearing aids. Despite technological advances and growing awareness of the health consequences of untreated hearing loss, adoption remains limited.

As highlighted in a comprehensive review, help-seeking and uptake are influenced more by psychosocial than demographic factors [22]. Our findings reinforce this perspective: neither age nor hearing loss status independently predicted intention once attitude was included in the model.

### Epidemiology and provision in Germany: structural availability versus utilization

Germany provides a particularly informative context because hearing aids are covered under statutory health insurance with defined reimbursement standards and regulated fitting procedures [20]. In addition, national quality assurance guidelines mandate structured indication, adaptation, verification, and outcome control processes [23]. From a structural perspective, barriers such as access and basic affordability are comparatively minimized.

Nevertheless, lifetime cost analyses demonstrate that out-of-pocket expenses and upgrade costs remain non-negligible for many patients [40]. However, in the present study, cost concerns—although frequently endorsed—showed only weak association with intention and did not significantly differentiate users from non-users. This pattern suggests that while financial considerations are salient at a descriptive level, they may not independently determine adoption once psychosocial variables are accounted for. Similar conclusions have been drawn in international reviews, where demographic and structural factors show inconsistent predictive value compared with psychosocial determinants [22].

Thus, the German context illustrates a critical distinction: structural provision does not automatically translate into utilization.

### Attitude as a central determinant of intention

In our cohort, the strong correlation between general attitude and behavioral intention (*r* = 0.63) and the substantial variance explained in regression (R² = 0.40) underscore the centrality of attitudinal factors in the decision-making process. This finding is consistent with the Theory of Reasoned Action framework, which posits that behavioral intention is primarily shaped by attitudes and subjective norms. Cobelli et al., demonstrated that attitude toward hearing aid adoption significantly predicts intention, whereas perceived social norms may negatively influence it [9]. Similarly, Meister et al., reported that expectations regarding quality-of-life improvement, stigmatization, and self-rated hearing ability explained 55% of the variance in willingness to use hearing aids [29]. A 2024 validation study on attitudes toward hearing aid use further confirmed the centrality of attitudinal constructs across working-age adults [42].

A German pilot study from 2004 identified psychosocial readiness, self-perception, and expectations as critical factors even before the formal provision process begins [28]. Subsequent German studies emphasized that non-audiological factors influence rehabilitation success after fitting [7]. These earlier German findings are consistent with contemporary international data, reinforcing the longitudinal stability of attitudinal determinants.

Our data extend these findings by showing that general attitude remains a dominant predictor even when controlling for age and hearing loss status. This suggests that cognitive-evaluative representations of hearing aids may outweigh clinical need in shaping behavioral intention.

### The role of stigma

In the present cohort, stigma-related beliefs emerged as one of the strongest correlates of intention (*r* = 0.65) and showed large between-group effects (d = − 0.78) between users and non-users. These findings align with the evidence that hearing loss and hearing aids are embedded in ageist and disability-related stereotypes [3]. Meyer et al., demonstrated that stigma-related experiences and concealment of hearing loss are associated with hearing aid use [30], and Ekberg et al., conceptualized disclosure versus concealment as central responses to stigma-induced identity threat [14].

Although explicit endorsement of stigma items in our sample was relatively low (e.g., embarrassment, unintelligence), domain-level stigma scores strongly predicted intention. This discrepancy reflects patterns observed in social representation research, where hearing aids are simultaneously associated with technological advancement and age-related decline [25].

Within the German system—where hearing aids are normalized within healthcare structures—stigma appears less as a structural barrier and more as an identity-level barrier. This distinction is crucial: system-level normalization does not eliminate symbolic meaning.

### Expected benefit and effort

The domain “expected benefit and effort” showed the strongest association with intention (*r* = 0.66) and large hearing aid user–non-user differences (d = − 0.75). This is highly consistent with prior evidence demonstrating that pre-fitting expectations significantly influence willingness and subsequent adoption [29]. In addition, Dwarakanath and Manjula reported that positive attitudes toward hearing loss and amplification were associated with greater use and perceived benefit and that negative attitudes toward hearing loss and hearing aids significantly increase the hazard of device abandonment over time [13]. Changes in attitudes have been shown to predict abandonment trajectories, even years after fitting [17, 39]. Taken together, these findings suggest that attitudes are not merely antecedents of uptake but dynamic determinants of sustained use.

The salience of this domain in our findings suggests that cognitive expectations regarding functionality and rehabilitation effort are central determinants of behavioral intention, potentially even more influential than cost concerns.

### Cost and social influence

Although cost concerns were the most strongly endorsed single barrier item, they showed only a weak correlation with intention (*r* = 0.18) and small, non-significant differences between users and non-users. This pattern is consistent with literature indicating that while cost is frequently cited as a barrier, it may not independently predict uptake once psychosocial variables are considered [22].

Similarly, social influence showed limited association with intention in our data. However, Cobelli et al., found that subjective norms negatively influence intention when individuals perceive disapproval from significant others [9]. It is possible that social norms operate more subtly or interactively rather than as direct predictors.

### Trust in physicians and health communication

Trust in physicians showed large group differences (d = − 0.87) between users and non-users but was not significantly correlated with intention in the full sample. Cobelli et al., reported that trust in health professionals mitigates negative social pressures and supports adoption [9]. Moreover, Adorni et al., demonstrated that the language used by physicians can significantly shape attitudes and intention toward hearing aid use [1]. Consumer studies in the US market show that individuals prefer in-person models and value professional guidance, even when over-the-counter options are available [37].

Our findings also reflect this and suggest that trust may play a more decisive role once hearing loss is acknowledged rather than at the level of general intention in the broader population. German quality assurance frameworks emphasize structured counseling and verification processes [23]. However, our findings suggest that technical adequacy alone is insufficient; attitudinal and identity-related concerns must be explicitly addressed during counseling.

### Limitations

The cross-sectional design precludes causal inference regarding the relationship between attitudes, intentions, and hearing aid use. Self-reported measures may be influenced by social desirability, particularly in stigma-related domains. While the study was sufficiently powered to detect effects of moderate magnitude, smaller associations may have gone undetected. Additionally, the heterogeneous sample may limit generalizability to clinical populations actively seeking hearing care.

Despite these limitations, understanding the mechanisms explored in this study is essential for designing targeted interventions to improve hearing aid uptake at the population level. Although conducted in a German-speaking population, the constructs examined are broadly applicable across healthcare contexts, providing insights that are relevant for international hearing health services research.

## Conclusion

Despite comprehensive structural provision and regulated quality assurance within the German healthcare system, hearing aid uptake remains strongly shaped by psychosocial determinants. Attitudes—particularly stigma-related beliefs and expectations regarding benefit and effort—emerged as central predictors of behavioral intention and differentiated users from non-users with large effect sizes. The adapted questionnaire demonstrated acceptable-to-good overall internal consistency, while associations with behavioral intention and hearing aid use provided preliminary evidence of construct validity. Exploratory factor analysis suggested a partially overlapping factor structure, indicating that further refinement and confirmatory psychometric evaluation are warranted. These findings support an integrated structural–psychosocial framework and underscore the necessity of addressing attitudinal determinants within hearing rehabilitation programs.

## Supplementary Information

Below is the link to the electronic supplementary material.

Supplementary Material 1

## Data Availability

The datasets generated and/or analyzed during the current study are not publicly available due to institutional data protection regulations but are available from the corresponding author on reasonable request.
