# Supplementary material for: Development and preliminary evaluation of a German questionnaire assessing attitudes toward hearing aids and behavioral intention
Source: BMC Health Serv Res. 2026 Sep 13;26:1274. doi: 10.1186/s12913-026-15549-0 (PMC13579736; doi:10.1186/s12913-026-15549-0)
Supplement: Supplementary file 1 [file 12913_2026_15549_MOESM1_ESM.docx]

**Supplement**

**Questionnaire in German language**

**ABSCHNITT 1**

**Stimmen Sie der Teilnahme an dieser Umfrage zu?**

- Ich stimme zu
- Ich stimme nicht zu

**Wie alt sind Sie?**

**Ihr Geschlecht:**

- Männlich
- Weiblich
- Divers

**Glauben Sie, dass Sie einen Hörverlust haben?**

- Ja
- Nein

*Bitte beantworten Sie die folgenden drei Fragen nur, wenn Sie die vorherige Frage mit* ***„Ja“*** *beantwortet haben.*

**Wie lange leiden Sie schon an Hörverlust?**

- Weniger als 1 Jahr
- 1-5 Jahre
- 6-10 Jahre
- Mehr als 10 Jahre

**Besitzen Sie derzeit Hörgeräte?**

- Ich besitze Hörgeräte und benutze sie
- Ich besitze Hörgeräte, aber ich benutze sie **nicht**
- Ich habe einen Hörverlust, aber ich besitze keine Hörgeräte

**Wie lange benutzen Sie schon ein Hörgerät?**

- Weniger als 6 Monate
- 6 Monate - 3 Jahre
- 3-6 Jahre
- Mehr als 6 Jahre

**ABSCHNITT 2**

**Erläuterungen**

Auf den folgenden Seiten finden Sie kurze Aussagen über Hörgeräte (z. B. „Mit einem Hörgerät würde ich mich alt fühlen“). Unter jeder Aussage finden Sie eine Bewertungsskala, auf der Sie auf einer 5-Punkte-Skala angeben können, ob Sie der Aussage zustimmen oder nicht zustimmen. Bitte kreuzen Sie das entsprechende Kästchen an, um anzugeben, wie sehr Sie der Aussage zustimmen oder nicht zustimmen (bzw. wie Sie handeln oder nicht handeln würden).

Zusätzlich gibt es eine zweite Skala, auf der gefragt wird, *welche Maßnahmen Sie* aufgrund Ihrer Antwort auf die vorhergehende Aussage *ergreifen würden*. Bitte kreuzen Sie das entsprechende Feld an, um Ihre Präferenz anzugeben.

| **Frage 1a. Ich glaube, die aktuell auf dem Markt verfügbaren Hörgeräte sind klein und unauffällig.** | | | | |
| --- | --- | --- | --- | --- |
| □  Stimme voll zu | □  Stimme eher zu | □  Keine Meinung | □  Stimme eher nicht zu | □  Stimme überhaupt nicht zu |
| **Frage 1b. Ausgehend von Ihrer Antwort auf die obige Aussage, welche Maßnahmen würden Sie ergreifen?** | | | | |
| □  Ich würde auf jeden Fall ein Hörgerät kaufen | □  Ich würde eher ein Hörgerät kaufen | □  Keine Meinung | □  Ich würde eher kein Hörgerät kaufen | □  Ich würde mir auf keinen Fall ein Hörgerät kaufen |

| **Frage 2a. Ich glaube, dass ein Hörgerät teuer ist.** | | | | |
| --- | --- | --- | --- | --- |
| □  Stimme überhaupt nicht zu | □  Stimme eher nicht zu | □  Keine Meinung | □  Stimme eher zu | □  Stimme voll zu |
| **Frage 2b. Ausgehend von Ihrer Antwort auf die obige Aussage, welche Maßnahmen würden Sie ergreifen?** | | | | |
| □  Ich würde auf jeden Fall ein Hörgerät kaufen | □  Ich würde eher ein Hörgerät kaufen | □  Keine Meinung | □  Ich würde eher kein Hörgerät kaufen | □  Ich würde mir auf keinen Fall ein Hörgerät kaufen |

| **Frage 3a. Ich glaube, ich komme mit meinem Hörverlust zurecht und brauche kein Hörgerät.** | | | | |
| --- | --- | --- | --- | --- |
| □  Stimme überhaupt nicht zu | □  Stimme eher nicht zu | □  Keine Meinung | □  Stimme eher zu | □  Stimme voll zu |
| **Frage 3b. Ausgehend von Ihrer Antwort auf die obige Aussage, welche Maßnahmen würden Sie ergreifen?** | | | | |
| □  Ich würde auf jeden Fall ein Hörgerät ausprobieren | □  Ich würde eher ein Hörgerät ausprobieren | □  Keine Meinung | □  Ich würde eher kein Hörgerät ausprobieren | □  Ich würde niemals ein Hörgerät ausprobieren |

| **Frage 4a. Ich glaube, dass ich mir ein optisch ansprechendes und unauffälliges Hörgerät nicht leisten kann.** | | | | |
| --- | --- | --- | --- | --- |
| □  Stimme überhaupt nicht zu | □  Stimme eher nicht zu | □  Keine Meinung | □  Stimme eher zu | □  Stimme voll zu |
| **Frage 4b. Ausgehend von Ihrer Antwort auf die obige Aussage, welche Maßnahmen würden Sie ergreifen?** | | | | |
| □  Ich würde auf jeden Fall ein Hörgerät kaufen | □  Ich würde eher ein Hörgerät kaufen | □  Keine Meinung | □  Ich würde eher kein Hörgerät kaufen | □  Ich würde mir auf keinen Fall ein Hörgerät kaufen |

| **Frage 5a. Ich erwarte, dass das Tragen eines Hörgeräts mein Gehör vollständig wiederherstellen würde.** | | | | |
| --- | --- | --- | --- | --- |
| □  Stimme voll zu | □  Stimme eher zu | □  Keine Meinung | □  Stimme eher nicht zu | □  Stimme überhaupt nicht zu |
| **Frage 5b. Ausgehend von Ihrer Antwort auf die obige Aussage, welche Maßnahmen würden Sie ergreifen?** | | | | |
| □  Ich würde auf jeden Fall ein Hörgerät ausprobieren | □  Ich würde eher ein Hörgerät ausprobieren | □  Keine Meinung | □  Ich würde eher kein Hörgerät ausprobieren | □  Ich würde niemals ein Hörgerät ausprobieren |

| **Frage 6a. Es wäre mir peinlich, wenn ich ein Hörgerät tragen würde.** | | | | |
| --- | --- | --- | --- | --- |
| □  Stimme voll zu | □  Stimme eher zu | □  Keine Meinung | □  Stimme eher nicht zu | □  Stimme überhaupt nicht zu |
| **Frage 6b. Ausgehend von Ihrer Antwort auf die obige Aussage, welche Maßnahmen würden Sie ergreifen?** | | | | |
| □  Ich würde auf jeden Fall ein Hörgerät tragen | □  Ich würde eher ein Hörgerät tragen | □  Keine Meinung | □  Ich würde eher kein Hörgerät tragen | □  Ich würde auf keinen Fall ein Hörgerät tragen |

| **Frage 7a. Ich ziehe ein Hörgerät in Betracht, um jemandem einen Gefallen zu tun (z.B. Ehepartner*in, Familienmitglied, Freund*in).** | | | | |
| --- | --- | --- | --- | --- |
| □  Stimme überhaupt nicht zu | □  Stimme eher nicht zu | □  Keine Meinung | □  Stimme eher zu | □  Stimme voll zu |
| **Frage 7b. Ausgehend von Ihrer Antwort auf die obige Aussage, welche Maßnahmen würden Sie ergreifen?** | | | | |
| □  Ich würde auf jeden Fall ein Hörgerät kaufen, um jemandem einen Gefallen zu tun | □  Ich würde eher ein Hörgerät kaufen, um jemandem einen Gefallen zu tun | □  Keine Meinung | □  Ich würde eher kein Hörgerät kaufen, um jemandem einen Gefallen zu tun | □  Ich würde auf keinen Fall ein Hörgerät kaufen, um jemandem einen Gefallen zu tun |

| **Frage 8a. Wenn ich ein Hörgerät trage, halten mich die Leute für dumm.** | | | | |
| --- | --- | --- | --- | --- |
| □  Stimme überhaupt nicht zu | □  Stimme eher nicht zu | □  Keine Meinung | □  Stimme eher zu | □  Stimme voll zu |
| **Frage 8b. Ausgehend von Ihrer Antwort auf die obige Aussage, welche Maßnahmen würden Sie ergreifen?** | | | | |
| □  Ich würde auf jeden Fall ein Hörgerät kaufen, unabhängig davon, was die Leute denken | □  Ich würde eher ein Hörgerät kaufen, unabhängig davon, was die Leute denken | □  Keine Meinung | □  Ich würde aus diesem Grund eher kein Hörgerät kaufen | □  Ich würde aus diesem Grund nie ein Hörgerät kaufen |

| **Frage 9a. Ich glaube, die Leute reagieren anders auf einen, wenn man ein Hörgerät trägt.** | | | | |
| --- | --- | --- | --- | --- |
| □  Stimme überhaupt nicht zu | □  Stimme eher nicht zu | □  Keine Meinung | □  Stimme eher zu | □  Stimme voll zu |
| **Frage 9b. Ausgehend von Ihrer Antwort auf die obige Aussage, welche Maßnahmen würden Sie ergreifen?** | | | | |
| □  Ich würde auf jeden Fall ein Hörgerät ausprobieren, egal wie die Leute reagieren | □  Ich würde eher ein Hörgerät ausprobieren, egal wie die Leute reagieren | □  Keine Meinung | □  Aus diesem Grund werde ich eher kein Hörgerät ausprobieren | □  Aus diesem Grund werde ich niemals ein Hörgerät ausprobieren |

| **Frage 10a. Mit einem Hörgerät würde ich in einer Menschenmenge auffallen.** | | | | |
| --- | --- | --- | --- | --- |
| □  Stimme überhaupt nicht zu | □  Stimme eher nicht zu | □  Keine Meinung | □  Stimme eher zu | □  Stimme voll zu |
| **Frage 10b. Ausgehend von Ihrer Antwort auf die obige Aussage, welche Maßnahmen würden Sie ergreifen?** | | | | |
| □  Ich würde auf jeden Fall ein Hörgerät tragen | □  Ich würde eher ein Hörgerät tragen | □  Keine Meinung | □  Ich würde eher kein Hörgerät tragen | □  Ich würde auf keinen Fall ein Hörgerät tragen |

| **Frage 11a. Die Leute wissen nicht, wie sie auf eine Person reagieren sollen, die ein Hörgerät trägt.** | | | | |
| --- | --- | --- | --- | --- |
| □  Stimme überhaupt nicht zu | □  Stimme eher nicht zu | □  Keine Meinung | □  Stimme eher zu | □  Stimme voll zu |
| **Frage 11b. Ausgehend von Ihrer Antwort auf die obige Aussage, welche Maßnahmen würden Sie ergreifen?** | | | | |
| □  Ich würde auf jeden Fall ein Hörgerät tragen | □  Ich würde eher ein Hörgerät tragen | □  Keine Meinung | □  Ich würde eher kein Hörgerät tragen | □  Ich würde auf keinen Fall ein Hörgerät tragen |
| **Frage 12a. Durch das Verwenden von Hörgeräten würde sich meine geistige Gesundheit verbessern.** | | | | |
| □  Stimme völlig zu | □  Stimme eher zu | □  Keine Meinung | □  Stimme eher nicht zu | □  Stimme überhaupt nicht zu |
| **Frage 12b. Ausgehend von Ihrer Antwort auf die obige Aussage, welche Maßnahmen würden Sie ergreifen?** | | | | |
| □  Ich würde auf jeden Fall einem Hörgerät eine Chance geben | □  Ich würde eher einem Hörgerät eine Chance geben | □  Keine Meinung | □  Ich würde einem Hörgerät eher keine Chance geben | □  Ich würde einem Hörgerät auf keinen Fall eine Chance geben |

| **Frage 13a. Ich glaube, es würde Wochen oder Monate dauern, bis ich mich an ein Hörgerät gewöhnt habe.** | | | | |
| --- | --- | --- | --- | --- |
| □  Stimme überhaupt nicht zu | □  Stimme eher nicht zu | □  Keine Meinung | □  Stimme eher zu | □  Stimme voll zu |
| **Frage 13b. Ausgehend von Ihrer Antwort auf die obige Aussage, welche Maßnahmen würden Sie ergreifen?** | | | | |
| □  Ich würde auf jeden Fall ein Hörgerät tragen | □  Ich würde eher ein Hörgerät tragen | □  Keine Meinung | □  Ich würde eher kein Hörgerät tragen | □  Ich würde auf keinen Fall ein Hörgerät tragen |

| **Frage 14a. Ich glaube, es ist kompliziert einen Kostenzuschlag für Hörgeräte durch die Krankenkasse zu beantragen.** | | | | |
| --- | --- | --- | --- | --- |
| □  Stimme überhaupt nicht zu | □  Stimme eher nicht zu | □  Keine Meinung | □  Stimme eher zu | □  Stimme voll zu |
| **Frage 14b. Ausgehend von Ihrer Antwort auf die obige Aussage, welche Maßnahmen würden Sie ergreifen?** | | | | |
| □  Ich würde auf jeden Fall ein Hörgerät ausprobieren | □  Ich würde eher ein Hörgerät ausprobieren | □  Keine Meinung | □  Aus diesem Grund würde ich eher kein Hörgerät ausprobieren | □  Aus diesem Grund würde ich niemals ein Hörgerät ausprobieren |

| **Frage 15a. Mit einem Hörgerät würde ich mich alt fühlen.** | | | | |
| --- | --- | --- | --- | --- |
| □  Stimme überhaupt nicht zu | □  Stimme eher nicht zu | □  Keine Meinung | □  Stimme eher zu | □  Stimme voll zu |
| **Frage 15b. Ausgehend von Ihrer Antwort auf die obige Aussage, welche Maßnahmen würden Sie ergreifen?** | | | | |
| □  Ich würde auf jeden Fall ein Hörgerät ausprobieren | □  Ich würde eher ein Hörgerät ausprobieren | □  Keine Meinung | □  Ich würde deshalb eher kein Hörgerät ausprobieren | □  Deshalb würde ich niemals ein Hörgerät ausprobieren |

| **Frage 16a. Ich wurde schon einmal durch meine*n Ärzt*in über Hörgeräte informiert.** | | | | |
| --- | --- | --- | --- | --- |
| □  Stimme völlig zu | □  Stimme eher zu | □  Keine Meinung | □  Stimme eher nicht zu | □  Stimme überhaupt nicht zu |
| **Frage 16b. Ausgehend von Ihrer Antwort auf die obige Aussage, welche Maßnahmen würden Sie ergreifen?** | | | | |
| □  Ich würde auf jeden Fall ein Hörgerät ausprobieren | □  Ich würde eher ein Hörgerät ausprobieren | □  Keine Meinung | □  Ich würde deshalb eher kein Hörgerät ausprobieren | □  Deshalb würde ich niemals ein Hörgerät ausprobieren |

| **Frage 17a. Ich fühle mich durch meine*n Ärzt*in gut über Hörgeräte beraten.** | | | | |
| --- | --- | --- | --- | --- |
| □  Stimme völlig zu | □  Stimme eher zu | □  Keine Meinung | □  Stimme eher nicht zu | □  Stimme überhaupt nicht zu |
| **Frage 17b. Ausgehend von Ihrer Antwort auf die obige Aussage, welche Maßnahmen würden Sie ergreifen?** | | | | |
| □  Ich würde mir auf jeden Fall ein Hörgerät besorgen | □  Ich würde mir eher ein Hörgerät besorgen | □  Keine Meinung | □  Ich würde mir eher kein Hörgerät besorgen | □  Ich würde mir auf keinen Fall ein Hörgerät besorgen |

| **Frage 18a. Soweit ich erfahren habe, sind Hörgeräte keine große Hilfe.** | | | | |
| --- | --- | --- | --- | --- |
| □  Stimme überhaupt nicht zu | □  Stimme eher nicht zu | □  Keine Meinung | □  Stimme eher zu | □  Stimme voll zu |
| **Frage 18b. Ausgehend von Ihrer Antwort auf die obige Aussage, welche Maßnahmen würden Sie ergreifen?** | | | | |
| □  Ich würde auf jeden Fall einer Probezeit mit Hörgeräten zustimmen | □  Ich würde eher ein Probezeit mit Hörgeräten zustimmen | □  Keine Meinung | □  Ich würde einer Probezeit mit Hörgeräten eher nicht zustimmen | □  Ich würde niemals einer Probezeit mit Hörgeräten zustimmen |

| **Frage 19a. Ich befürchte, dass die Krankenkasse die Kosten für meine Hörgeräte nicht übernimmt.** | | | | |
| --- | --- | --- | --- | --- |
| □  Stimme überhaupt nicht zu | □  Stimme eher nicht zu | □  Keine Meinung | □  Stimme eher zu | □  Stimme voll zu |
| **Frage 19b. Ausgehend von Ihrer Antwort auf die obige Aussage, welche Maßnahmen würden Sie ergreifen?** | | | | |
| □  Ich würde auf jeden Fall auch auf eigene Kosten ein Hörgerät kaufen | □  Ich würde mir eher auch auf eigene Kosten ein Hörgerät kaufen | □  Keine Meinung | □  Ich würde eher nicht auf eigene Kosten ein Hörgerät kaufen | □  Ich würde mir niemals auf eigene Kosten ein Hörgerät kaufen |

| **Frage 20a. Ich glaube, dass die Krankenkasse die Kosten für meine Hörgeräte übernimmt, wenn ich diese ärztlich verschrieben bekomme.** | | | | |
| --- | --- | --- | --- | --- |
| □  Stimme völlig zu | □  Stimme eher zu | □  Keine Meinung | □  Stimme eher nicht zu | □  Stimme überhaupt nicht zu |
| **Frage 20b. Ausgehend von Ihrer Antwort auf die obige Aussage, welche Maßnahmen würden Sie ergreifen?** | | | | |
| □  Ich würde mir auf jeden Fall ein Hörgerät verschreiben lassen | □  Ich würde mir eher ein Hörgerät verschreiben lassen | □  Keine Meinung | □  Ich würde mir eher kein Hörgerät verschreiben lassen | □  Ich würde mir niemals ein Hörgerät verschreiben lassen |

| **Frage 21a. Ich wäre bereit, ein Hörgerät auszuprobieren, aber ich glaube nicht, dass es eine große Hilfe sein würde.** | | | | |
| --- | --- | --- | --- | --- |
| □  Stimme überhaupt nicht zu | □  Stimme eher nicht zu | □  Keine Meinung | □  Stimme eher zu | □  Stimme voll zu |
| **Frage 21b. Ausgehend von Ihrer Antwort auf die obige Aussage, welche Maßnahmen würden Sie ergreifen?** | | | | |
| □  Ich würde auf jeden Fall einem Hörgerät eine Chance geben | □  Ich würde eher einem Hörgerät eine Chance geben | □  Keine Meinung | □  Ich würde einem Hörgerät eher keine Chance geben | □  Ich würde einem Hörgerät auf keinen Fall eine Chance geben |

**Questionnaire in English language**

**SECTION 1**

**Do you agree to participate in this survey?**

- I agree
- I do not agree

**What is your current age?**

**Your sex:**

- Male
- Female
- Diverse

**Do you think you have hearing loss?**

- Yes
- No

*Please answer the following three questions only if you answered “Yes” to the previous question.*

**How long have you experienced hearing loss?**

- Less than 1 year
- 1 - 5 years
- 6 - 10 years
- More than 10 years

**Do you currently use hearing aids?**

- I have hearing aids and use them
- I have hearing aids but do not use them
- I do not have or use hearing aids

**How long have you been using a hearing aid?**

- Less than 6 months
- 6 months - 3 years
- 3 - 6 years
- More than 6 years

**SECTION 2**

**Instructions**

On the following pages you will find short statements about hearing aids (e.g., “Wearing a hearing aid would make me feel old”). Under each statement you will find a rating scale on which you can indicate, using a 5‑point scale, whether you agree or disagree with the statement. Please tick the appropriate box to indicate how strongly you agree or disagree with the statement (or how you would or would not act). Each statement is followed by a second question asking what action you would take based on your response to that statement.

| **Q1a. I believe the hearing aids currently available on the market are small and discreet.** | | | | |
| --- | --- | --- | --- | --- |
| □  Strongly agree | □  Somewhat agree | □  No opinion | □  Somewhat disagree | □  Strongly disagree |
| **Q1b. Based on your answer to the previous statement, what action would you take?** | | | | |
| □  I would definitely buy a hearing aid | □  I would probably buy a hearing aid | □  No opinion | □  I would probably not buy a hearing aid | □  I would never buy a hearing aid |

| **Q2a. I believe that a hearing aid is expensive.** | | | | |
| --- | --- | --- | --- | --- |
| □  Strongly disagree | □  Somewhat disagree | □  No opinion | □  Somewhat agree | □  Strongly agree |
| **Q2b. Based on your answer to the previous statement, what action would you take?** | | | | |
| □  I would definitely buy a hearing aid | □  I would probably buy a hearing aid | □  No opinion | □  I would probably not buy a hearing aid | □  I would never buy a hearing aid |

| **Q3a. I believe I can cope with my hearing loss and do not need a hearing aid.** | | | | |
| --- | --- | --- | --- | --- |
| □  Strongly disagree | □  Somewhat disagree | □  No opinion | □  Somewhat agree | □  Strongly agree |
| **Q3b. Based on your answer to the previous statement, what action would you take?** | | | | |
| □  I would definitely try a hearing aid | □  I would probably try a hearing aid | □  No opinion | □  I would probably not try a hearing aid | □  I would never try a hearing aid |

| **Q4a. I believe I cannot afford a small and discreet hearing aid.** | | | | |
| --- | --- | --- | --- | --- |
| □  Strongly disagree | □  Somewhat disagree | □  No opinion | □  Somewhat agree | □  Strongly agree |
| **Q4b. Based on your answer to the previous statement, what action would you take?** | | | | |
| □  I would definitely buy a hearing aid | □  I would probably buy a hearing aid | □  No opinion | □  I would probably not buy a hearing aid | □  I would never buy a hearing aid |

| **Q5a. I expect that wearing a hearing aid would completely restore my hearing.** | | | | |
| --- | --- | --- | --- | --- |
| □  Strongly agree | □  Somewhat agree | □  No opinion | □  Somewhat disagree | □  Strongly disagree |
| **Q5b. Based on your answer to the previous statement, what action would you take?** | | | | |
| □  I would definitely try a hearing aid | □  I would probably try a hearing aid | □  No opinion | □  I would probably not try a hearing aid | □  I would never try a hearing aid |

| **Q6a. I would be embarrassed if I wore a hearing aid.** | | | | |
| --- | --- | --- | --- | --- |
| □  Strongly agree | □  Somewhat agree | □  No opinion | □  Somewhat disagree | □  Strongly disagree |
| **Q6b. Based on your answer to the previous statement, what action would you take?** | | | | |
| □  I would definitely wear a hearing aid | □  I would probably wear a hearing aid | □  No opinion | □  I would probably not wear a hearing aid | □  I would never wear a hearing aid |

| **Q7a. I would consider using a hearing aid to please someone else (e.g., my spouse, family member, or friend).** | | | | |
| --- | --- | --- | --- | --- |
| □  Strongly disagree | □  Somewhat disagree | □  No opinion | □  Somewhat agree | □  Strongly agree |
| **Q7b. Based on your answer to the previous statement, what action would you take?** | | | | |
| □  I would definitely buy a hearing aid | □  I would probably buy a hearing aid | □  No opinion | □  I would probably not buy a hearing aid | □  I would never buy a hearing aid |

| **Q8a. If I wear a hearing aid, others will perceive me as less intelligent.** | | | | |
| --- | --- | --- | --- | --- |
| □  Strongly disagree | □  Somewhat disagree | □  No opinion | □  Somewhat agree | □  Strongly agree |
| **Q8b. Based on your answer to the previous statement, what action would you take?** | | | | |
| □  I would definitely buy a hearing aid regardless of what people think | □  I would probably buy a hearing aid regardless of what people think | □  No opinion | □  I would probably not buy a hearing aid for this reason | □  I would never buy a hearing aid for this reason |

| **Q9a. I believe people react differently to someone who wears a hearing aid.** | | | | |
| --- | --- | --- | --- | --- |
| □  Strongly disagree | □  Somewhat disagree | □  No opinion | □  Somewhat agree | □  Strongly agree |
| **Q9b. Based on your answer to the previous statement, what action would you take?** | | | | |
| □  I would definitely try a hearing aid, regardless of others’ reactions | □  I would probably try a hearing aid,  regardless of others’ reactions | □  No opinion | □  For this reason I would probably not try a hearing aid | □  For this reason  I would never try a hearing aid |

| **Q10a. Wearing a hearing aid would make me stand out in a crowd.** | | | | |
| --- | --- | --- | --- | --- |
| □  Strongly disagree | □  Somewhat disagree | □  No opinion | □  Somewhat agree | □  Strongly agree |
| **Q10b. Based on your answer to the previous statement, what action would you take?** | | | | |
| □  I would definitely wear a hearing aid | □  I would probably wear a hearing aid | □  No opinion | □  I would probably not wear a hearing aid | □  I would never wear a hearing aid |

| **Q11a. People often do not know how to interact with someone who wears a hearing aid.** | | | | |
| --- | --- | --- | --- | --- |
| □  Strongly disagree | □  Somewhat disagree | □  No opinion | □  Somewhat agree | □  Strongly agree |
| **Q11b. Based on your answer to the previous statement, what action would you take?** | | | | |
| □  I would definitely wear a hearing aid | □  I would probably wear a hearing aid | □  No opinion | □  I would probably not wear a hearing aid | □  I would never wear a hearing aid |

| **Q12a. Using hearing aids would improve my emotional and mental well-being.** | | | | |
| --- | --- | --- | --- | --- |
| □  Strongly agree | □  Somewhat agree | □  No opinion | □  Somewhat disagree | □  Strongly disagree |
| **Q12b. Based on your answer to the previous statement, what action would you take?** | | | | |
| □  I would definitely try a hearing aid | □  I would probably try a hearing aid | □  No opinion | □  I would probably not try a hearing aid | □  I would never try a hearing aid |

| **Q13a. I believe it would take weeks or months to get used to a hearing aid.** | | | | |
| --- | --- | --- | --- | --- |
| □  Strongly disagree | □  Somewhat disagree | □  No opinion | □  Somewhat agree | □  Strongly agree |
| **Q13b. Based on your answer to the previous statement, what action would you take?** | | | | |
| □  I would definitely wear a hearing aid | □  I would probably wear a hearing aid | □  No opinion | □  I would probably not wear a hearing aid | □  I would never wear a hearing aid |

| **Q14a. I believe it is complicated to apply for health‑insurance reimbursement for hearing aids.** | | | | |
| --- | --- | --- | --- | --- |
| □  Strongly disagree | □  Somewhat disagree | □  No opinion | □  Somewhat agree | □  Strongly agree |
| **Q14b. Based on your answer to the previous statement, what action would you take?** | | | | |
| □  I would definitely try a hearing aid | □  I would probably try a hearing aid | □  No opinion | □  I would probably not try a hearing aid | □  I would never try a hearing aid |

| **Q15a. Wearing a hearing aid would make me feel old.** | | | | |
| --- | --- | --- | --- | --- |
| □  Strongly disagree | □  Somewhat disagree | □  No opinion | □  Somewhat agree | □  Strongly agree |
| **Q15b. Based on your answer to the previous statement, what action would you take?** | | | | |
| □  I would definitely try a hearing aid | □  I would probably try a hearing aid | □  No opinion | □  I would probably not try a hearing aid | □  I would never try a hearing aid |

| **Q16a. My physician has previously informed me about hearing aids.** | | | | |
| --- | --- | --- | --- | --- |
| □  Strongly agree | □  Somewhat agree | □  No opinion | □  Somewhat disagree | □  Strongly disagree |
| **Q16b. Based on your answer to the previous statement, what action would you take?** | | | | |
| □  I would definitely try a hearing aid | □  I would probably try a hearing aid | □  No opinion | □  I would probably not try a hearing aid | □  I would never try a hearing aid |

| **Q17a. I feel that my doctor has provided adequate information about hearing aids.** | | | | |
| --- | --- | --- | --- | --- |
| □  Strongly agree | □  Somewhat agree | □  No opinion | □  Somewhat disagree | □  Strongly disagree |
| **Q17b. Based on your answer to the previous statement, what action would you take?** | | | | |
| □  I would definitely buy a hearing aid | □  I would probably buy a hearing aid | □  No opinion | □  I would probably not buy a hearing aid | □  I would never buy a hearing aid |

| **Q18a. From what I have heard, hearing aids are not very helpful.** | | | | |
| --- | --- | --- | --- | --- |
| □  Strongly disagree | □  Somewhat disagree | □  No opinion | □  Somewhat agree | □  Strongly agree |
| **Q18b. Based on your answer to the previous statement, what action would you take?** | | | | |
| □  I would definitely try a hearing aid | □  I would probably try a hearing aid | □  No opinion | □  I would probably not try a hearing aid | □  I would never try a hearing aid |

| **Q19a. I am concerned that my health insurance will not cover the cost of my hearing aids.** | | | | |
| --- | --- | --- | --- | --- |
| □  Strongly disagree | □  Somewhat disagree | □  No opinion | □  Somewhat agree | □  Strongly agree |
| **Q19b. Based on your answer to the previous statement, what action would you take?** | | | | |
| □  I would definitely buy a hearing aid even at my own expense | □  I would probably buy a hearing aid even at my own expense | □  No opinion | □  I would probably not buy a hearing aid if the cost were not covered. | □  I would probably not buy a hearing aid if the cost were not covered. |

| **Q20a. I believe that my health insurance will cover the cost of my hearing aids if they are prescribed by a doctor.** | | | | |
| --- | --- | --- | --- | --- |
| □  Strongly agree | □  Somewhat agree | □  No opinion | □  Somewhat disagree | □  Strongly disagree |
| **Q20b. Based on your answer to the previous statement, what action would you take?** | | | | |
| □  I would definitely get a prescription for a hearing aid | □  I would probably get a prescription for a hearing aid | □  No opinion | □  I would probably not get a prescription for a hearing aid | □  I would never get a prescription for a hearing aid |

| **Q21a. I would be willing to try a hearing aid, but I do not believe it would be very helpful.** | | | | |
| --- | --- | --- | --- | --- |
| □  Strongly disagree | □  Somewhat disagree | □  No opinion | □  Somewhat agree | □  Strongly agree |
| **Q21b. Based on your answer to the previous statement, what action would you take?** | | | | |
| □  I would definitely try a hearing aid | □  I would probably try a hearing aid | □  No opinion | □  I would probably not try a hearing aid | □  I would never try a hearing aid |

**Supplementary Figure S1. Scree plot of eigenvalues from the exploratory factor analysis.**

*Legend*

*Scree plot of eigenvalues obtained from the exploratory factor analysis of the 20-item attitude questionnaire. Visual inspection demonstrates a marked decline after the first factor followed by progressive leveling of the curve. Together with the Kaiser criterion (four eigenvalues >1) and interpretability of the rotated solution, these findings supported retention of a four-factor solution.*

**Supplementary Table S1. Pattern matrix from exploratory factor analysis of the 20-item attitude questionnaire**

| Questionnaire item | Factor 1 | Factor 2 | Factor 3 | Factor 4 | Uniqueness |
| --- | --- | --- | --- | --- | --- |
| Q1a. Hearing aids currently available are small and discreet. | – | – | – | **0.379** | 0.835 |
| Q2a. Hearing aids are expensive. | – | – | – | **−0.396** | 0.796 |
| Q4a. I cannot afford a small and discreet hearing aid. | **0.324** | – | – | – | 0.746 |
| Q5a. Hearing aids would completely restore my hearing. | **0.537** | – | **−0.517** | – | 0.617 |
| Q6a. I would be embarrassed if I wore a hearing aid. | **0.568** | – | – | – | 0.418 |
| Q7a. I would use a hearing aid to please someone else. | – | – | – | – | 0.871 |
| Q8a. Others would perceive me as less intelligent. | **0.406** | **0.491** | – | – | 0.482 |
| Q9a. People react differently to someone wearing a hearing aid. | **0.327** | – | – | **−0.518** | 0.570 |
| Q10a. Wearing a hearing aid would make me stand out. | **0.480** | – | – | – | 0.593 |
| Q11a. People do not know how to interact with hearing-aid users. | – | **0.338** | – | **−0.445** | 0.537 |
| Q12a. Hearing aids improve emotional and mental well-being. | – | – | – | **0.475** | 0.684 |
| Q13a. It takes weeks or months to get used to hearing aids. | **0.670** | – | – | – | 0.577 |
| Q14a. Applying for reimbursement is complicated. | – | – | **0.681** | – | 0.563 |
| Q15a. Wearing a hearing aid would make me feel old. | **0.318** | – | **0.322** | – | 0.618 |
| Q16a. My physician has informed me about hearing aids. | – | **−0.741** | – | – | 0.487 |
| Q17a. My doctor has provided adequate information. | – | **−0.692** | – | – | 0.562 |
| Q18a. Hearing aids are not very helpful. | – | **0.470** | – | – | 0.535 |
| Q19a. Insurance will not cover hearing aids. | – | – | **0.660** | – | 0.550 |
| Q20a. Insurance covers prescribed hearing aids. | – | – | – | **0.512** | 0.690 |
| Q21a. I would try hearing aids, but they would not be helpful. | **0.634** | – | – | – | 0.589 |

*Legend*

*Exploratory factor analysis was performed using principal axis factoring with Promax (oblique) rotation. One item ("I believe I can cope with my hearing loss and do not need a hearing aid.") was excluded because it was only applicable to participants with self-reported hearing loss. Four factors were retained based on eigenvalues >1, inspection of the scree plot, and interpretability of the rotated solution. Only factor loadings with an absolute value ≥0.30 are displayed. Items loading ≥0.30 on more than one factor are considered to demonstrate cross-loadings. Uniqueness represents the proportion of variance not explained by the retained factors.*

**Supplementary Table S2. Item-level internal consistency statistics for the 20-item attitude questionnaire**

| Questionnaire item | Corrected item–total correlation | Cronbach's α if item deleted |
| --- | --- | --- |
| **Q1a. Hearing aids currently available are small and discreet.** | **0.111** | **0.767** |
| **Q2a. Hearing aids are expensive.** | **0.164** | **0.765** |
| **Q4a. I cannot afford a small and discreet hearing aid.** | **0.395** | **0.750** |
| **Q5a. Hearing aids would completely restore my hearing.** | **0.066** | **0.771** |
| **Q6a. I would be embarrassed if I wore a hearing aid.** | **0.577** | **0.735** |
| **Q7a. I would use a hearing aid to please someone else.** | **0.144** | **0.769** |
| **Q8a. Others would perceive me as less intelligent.** | **0.570** | **0.739** |
| **Q9a. People react differently to someone wearing a hearing aid.** | **0.341** | **0.753** |
| **Q10a. Wearing a hearing aid would make me stand out.** | **0.487** | **0.744** |
| **Q11a. People do not know how to interact with hearing-aid users.** | **0.494** | **0.744** |
| **Q12a. Hearing aids improve emotional and mental well-being.** | **−0.027** | **0.780** |
| **Q13a. It takes weeks or months to get used to hearing aids.** | **0.251** | **0.761** |
| **Q14a. Applying for reimbursement is complicated.** | **0.307** | **0.757** |
| **Q15a. Wearing a hearing aid would make me feel old.** | **0.422** | **0.748** |
| **Q16a. My physician has informed me about hearing aids.** | **0.271** | **0.759** |
| **Q17a. My doctor has provided adequate information.** | **0.285** | **0.759** |
| **Q18a. Hearing aids are not very helpful.** | **0.491** | **0.744** |
| **Q19a. Insurance will not cover hearing aids.** | **0.397** | **0.749** |
| **Q20a. Insurance covers prescribed hearing aids.** | **0.111** | **0.766** |
| **Q21a. I would try a hearing aid, but it would not be helpful.** | **0.391** | **0.751** |

*Legend*

*Corrected item–total correlations were calculated using the total attitude score excluding the respective item. Cronbach's α if item deleted represents the estimated internal consistency of the overall attitude scale following removal of the corresponding item.*

**Supplementary Table S3.**

**Descriptive Statistics for Individual Attitudinal Items**

| Item | Mean | SD | N |
| --- | --- | --- | --- |
| 1a* | 1.90 | 0.93 | 156 |
| 2a | 3.85 | 0.98 | 156 |
| 3a | 3.03 | 1.31 | 113 |
| 4a | 3.05 | 1.24 | 156 |
| 5a* | 2.51 | 1.13 | 158 |
| 6a | 2.18 | 1.25 | 158 |
| 7a | 2.66 | 1.47 | 157 |
| 8a | 1.63 | 1.11 | 157 |
| 9a | 2.89 | 1.30 | 156 |
| 10a | 2.26 | 1.15 | 156 |
| 11a | 2.44 | 1.14 | 156 |
| 12a* | 2.51 | 1.23 | 154 |
| 13a | 3.30 | 1.11 | 154 |
| 14a | 3.01 | 1.22 | 153 |
| 15a | 2.45 | 1.33 | 155 |
| 16a* | 3.63 | 1.65 | 153 |
| 17a* | 3.05 | 1.30 | 139 |
| 18a | 2.00 | 1.13 | 157 |
| 19a | 3.03 | 1.26 | 155 |
| 20a* | 2.15 | 0.87 | 153 |
| 21a | 2.49 | 1.23 | 149 |

*Legend*

*Items were rated on 5-point Likert scales (1 = strongly disagree to 5 = strongly agree). *Items were rated on 5-point Likert scales (1 = strongly agree to 5 = strongly disagree, so that the direction of all the questions is the same.*
